# AMPK Activation by A-769662 Controls IL-6 Expression in Inflammatory Arthritis

**DOI:** 10.1371/journal.pone.0140452

**Published:** 2015-10-16

**Authors:** Monica Guma, Yun Wang, Benoit Viollet, Ru Liu-Bryan

**Affiliations:** 1 Division of Rheumatology, Allergy and Immunology, UC San Diego School of Medicine, La Jolla, California, United States of America; 2 INSERM, U1016, Institut Cochin, Paris, France; 3 CNRS, UMR8104, Paris, France; 4 Université Paris Descartes, Sorbonne Paris Cité, Paris, France; 5 VA San Diego Healthcare System, 3350 La Jolla Village Drive, La Jolla, California, United States of America; Université catholique de Louvain, BELGIUM

## Abstract

**Objective:**

AMP-activated protein kinase (AMPK) is a serine/threonine protein kinase critically involved in the regulation of cellular energy homeostasis. It is a central regulator of both lipid and glucose metabolism. Many studies have suggested that AMPK activation exert significant anti-inflammatory and immunosuppressive effects. In this study, we assessed whether targeted activation of AMPK inhibits inflammatory arthritis *in vivo*.

**Methods:**

We tested the effect of A-769662, a specific AMPK agonist (60mg/kg/bid) in mouse models of antigen-induced arthritis (AIA) and passive K/BxN serum-induced arthritis. The passive K/BxN serum-induced arthritis model was also applied to AMPKα1-deficient mice. Joints were harvested and subjected to histological analysis. IL-6 expression was measured in both joint tissues and sera by ELISA. The effect of A-769662 on bone marrow derived macrophage (BMDM) response to stimulation with TLR2 and TLR4 agonists was tested *in vitro*.

**Results:**

AMPK activation by A-769662 reduced inflammatory infiltration and joint damage in both mouse models. IL-6 expression in serum and arthritic joints was significantly decreased in A-769662-treated mice. AMPKα1 deficient mice mildly elicited an increase of clinical arthritis. IL-6 expression at both mRNA and protein levels, phosphorylation of p65 NF-κB and MAPK phosphorylation were inhibited by A-769662 in BMDMs stimulated with either TLR2 or TLR4 agonists.

**Conclusions:**

AMPK activation by specific AMPK agonist A-769662 suppressed inflammatory arthritis in mice as well as IL-6 expression in serum and arthritic joints. These data suggest that targeted activation of AMPK has a potential to be an effective therapeutic strategy for IL-6 dependent inflammatory arthritis.

## Introduction

AMPK is a crucial regulator of energy metabolic homeostasis at the cellular and whole body levels [1]. AMPK is a serine/threonine kinase that consists of a heterotrimeric complex including a catalytic α subunit and regulatory β and γ units [1–3]. There are two isoforms of α (α1 and α2) and β (β1and β2) and three γ subunits (γ1–3), which are differently expressed in mammalian tissues [1–3]. AMPK is activated via allosteric regulation of increased AMP concentration and by the phosphorylation of α subunit (Thr172) via several upstream kinases [1–3]. The AMPK signaling plays a key role in cellular and organismal survival during stress by its ability to maintain metabolic homeostasis. Interestingly, there is emerging evidence that activation of AMPK can suppress activation of nuclear factor-κB (NF-κB), a key regulator of innate immunity and inflammation [3].

The transcription factor NF-κB has been well recognized as a pivotal regulator of inflammation in rheumatoid arthritis (RA) [4, 5]. RA is a disabling chronic inflammatory autoimmune disease affecting synovial joints [6]. Experimental evidence suggests that NF-κB activation plays a pivotal role both at the stage of initiation and the stage of perpetuation of chronic inflammation in RA, including development of T helper 1 responses, activation, abnormal apoptosis and proliferation of RA fibroblast-like synovial cells, and differentiation and activation of bone resorbing activity of osteoclasts. In agreement with this, studies in animal models of RA have demonstrated the high therapeutic efficacy of specific inhibitors of NF-κB signaling pathway [7].

Activation of AMPK can be achieved by pharmacological activators such as AICAR and A-769662. AICAR activates AMPK by increasing cellular ZMP (a homologue of AMP):ATP ratio. It has been extensively used in multiple cell lines and some autoimmune diseases [8–10]. However, it suffers from unfavorable pharmacokinetic properties (high effective concentration, poor bioavailability and short half life), severe metabolic complications (e.g., lactic acidosis and massive uric acid production) and lack of a strict specificity for AMPK as it interacts with other enzymes, such as S-adenosylhomocysteine hydrolase and glycogen phosphorylase. In comparison, A-769662 is a specific and direct AMPK activator by binding directly to AMPK β1 subunit at a site distinct from the nucleotides, causing a direct allosteric activation and inhibition of dephosphorylation [11].

In this study, we tested the effect of A-769662 in inflammatory arthritis in mice. We found that AMPK activation by A-769662 reduced inflammatory infiltration and joint damage in two different animal models of inflammatory arthritis. In addition, IL-6 expression in serum and arthritic joints was significantly decreased in A-769662 treated mice. Moreover, AMPK activation in BMDMs by A-769662 reduced IL-6 expression and secretion, phosphorylation of p65 NF-κB (ser536) and phosphorylation of ERK and p38 MAPKs in response to TLR4 agonist LPS. Our data suggest that direct activation of AMPK could be potentially used as a therapeutic option in inflammatory arthritis.

## Material and Methods

### Mice

KRN T cell receptor (TCR) transgenic mice on the C57BL/6 background were a gift from Drs. D. Mathis and C. Benoist (Harvard Medical School, Boston, MA) and Institut de Génétique et de Biologie Moléculaire et Cellulaire (Strasbourg, France). AMPKα1 KO mice were generated by Dr. Benoit Viollet’s group. *Ampkα1* ^*+/+*^ and *Ampkα1* ^*−/−*^ mice in C57BL/6×129/Sv mixed background were used. Serum transfer and antigen induced arthritis models were induced in C57BL/6 background mice. Mice used in these experiments were 8–12 weeks old. All animal protocols received prior approval by the University of California at San Diego Institutional Animal Care and Use Committee (IACUC) and follow the NIH Guide for the Care and Use of Laboratory Animals.

### Preparation of BMDMs

To generate BMDMs, bone marrow cells from C57BL/6 background mice, were cultured in DMEM (Invitrogen) with 10% FBS and 20% L929 supernatant containing macrophage-stimulating factor for 6 days and were replated for the assays as indicated. *Ampkα1* ^*+/+*^ and *Ampkα1* ^*−/−*^ BMDMs were generated from a C57BL/6×129/Sv mixed background.

### Reagents

A-769662 was purchased from LC Labs (Woburn, MA) Lipopolysaccharide (LPS; *Escherichia coli* 0111:B4) and Pam3C were purchased from Sigma (St. Louis, MO).

### Real-time quantitative PCR (qPCR)

BMDMs and joints were collected. Joints were dissected to remove extra-articular tissue, and snap frozen in liquid nitrogen. The specimens were then pulverized. Total RNA was extracted with Trizol (Invitrogen) and reverse-transcribed with random hexamers and Superscript II Kit (Invitrogen). qPCR was performed with SYBR Green PCR Master Mix Kit (Applied Biosystems, Foster City, CA). The relative amounts of transcripts were compared to those of HRPT and normalized to untreated samples by the ^ΔΔ^Ct method. Primer sequences are available upon request.

### Western blot analysis

BMDMs and mouse joint tissues were disrupted in lysis buffer (PhosphoSafe™, Novagen, Gibbstown, NJ) containing a protease inhibitor cocktail. Proteins were separated by SDS-PAGE and transferred to a nitrocellulose membrane. Blots were probed with antibodies against phospho-AMPKα (Thr172), total AMPKα, phospho-p65 (Ser536), total p65, phospho-p38 (Thr180/Tyr182), total p38, phospho-ERK (Thr202/Tyr204), total ERK, phospho-ACC (Ser79) and total ACC (Cell Signaling Technology, Denvers, MA). Horseradish peroxidase-conjugated anti-IgG (Cell Signaling Technology, Danvers, MA) was used as secondary antibody. Membranes were developed using a chemiluminescence system (ECL detection reagent: Amersham Life Science, Aylesbury, UK). Densitometry analysis was done using Quantity One 1-D analysis software (Bio-Rad).

### Cytokine and nitric oxide (NO) quantification

The conditioned media was used to measure release of IL-1β and IL-6, and NO by DuoSet enzyme-linked immunosorbent assay (ELISA; R&D Systems, Minneapolis, MN) and Griess method (Cayman Chemicals, Ann Arbor, MI), respectively, following manufacturers’ protocol.

### Serum transfer and arthritis scoring

Sera from arthritic adult K/BxN mice were pooled and recipient mice were injected intraperitoneally (i.p.) with 150 μl of K/BxN serum on day 0. A-769662 (30 and 60mg/kg/bid) was injected daily i.p. beginning on day 0. Control group was injected with the same amount of DMSO as needed to dissolve the mentioned drugs. Clinical arthritis scores were evaluated as described [12].

### Antigen-induced arthritis (AIA) induction

Experimental AIA was induced by injection of 100 μg of methylated bovine serum albumin (mBSA) emulsified in 100 μl of complete Freund’s adjuvant (CFA) subcutaneously (s.c.) in the flank and then another injection one week later of 100 μg of mBSA/CFA intradermally (i.d.) in the tail base. Two weeks after these injections, arthritis was induced by intraarticular injection of 60 μg of mBSA in 10 μl of saline into the right knee joint. The left knee was injected with saline or PBS to serve as a control. A-769662 (60mg/kg/bid) was injected daily intraperitoneally (i.p.) beginning on day 0 or day 5 after intraarticular injection. Disease was assessed 10 days post-intraarticular injection by histological analysis as described below.

### Histology analysis

Joints were fixed in 10% formalin, decalcified in formic acid for 3 days, and paraffin embedded. Sections were prepared from the tissue blocks and stained with hematoxylin and eosin (H&E) and safranin O. A blinded semiquantitative scoring system was used to assess synovial inflammation, bone erosion and cartilage damage (0–4 scale), as previously described [12].

### Determination of serum antibodies

Methylated BSA-specific IgG1 and IgG2b antibodies were measured in sera of individuals by ELISA. Antigen was coated on microtiter plates at a concentration of 10 μg/ml. Antibody titers were assessed by 2-fold serial dilutions of sera, followed by detection of bound mouse Ig with a 1:500 dilution of peroxidase-conjugated rabbit anti-mouse Ig. O-phenylenediamine was used as substrate for the peroxidase reactions.

### Statistical analysis

Data are expressed as mean ± standard error of the mean (SEM). Mann Whitney U test was used for pair-wise comparisons and ANOVA for multiple group comparisons. All statistical analyses were performed using PRISM version 4.0b (GraphPad Software, San Diego, California). Results were considered significant for p<0.05.

## Results

### Effect of AMPK activation by A-769662 on BMDM function

**Fig 1:**
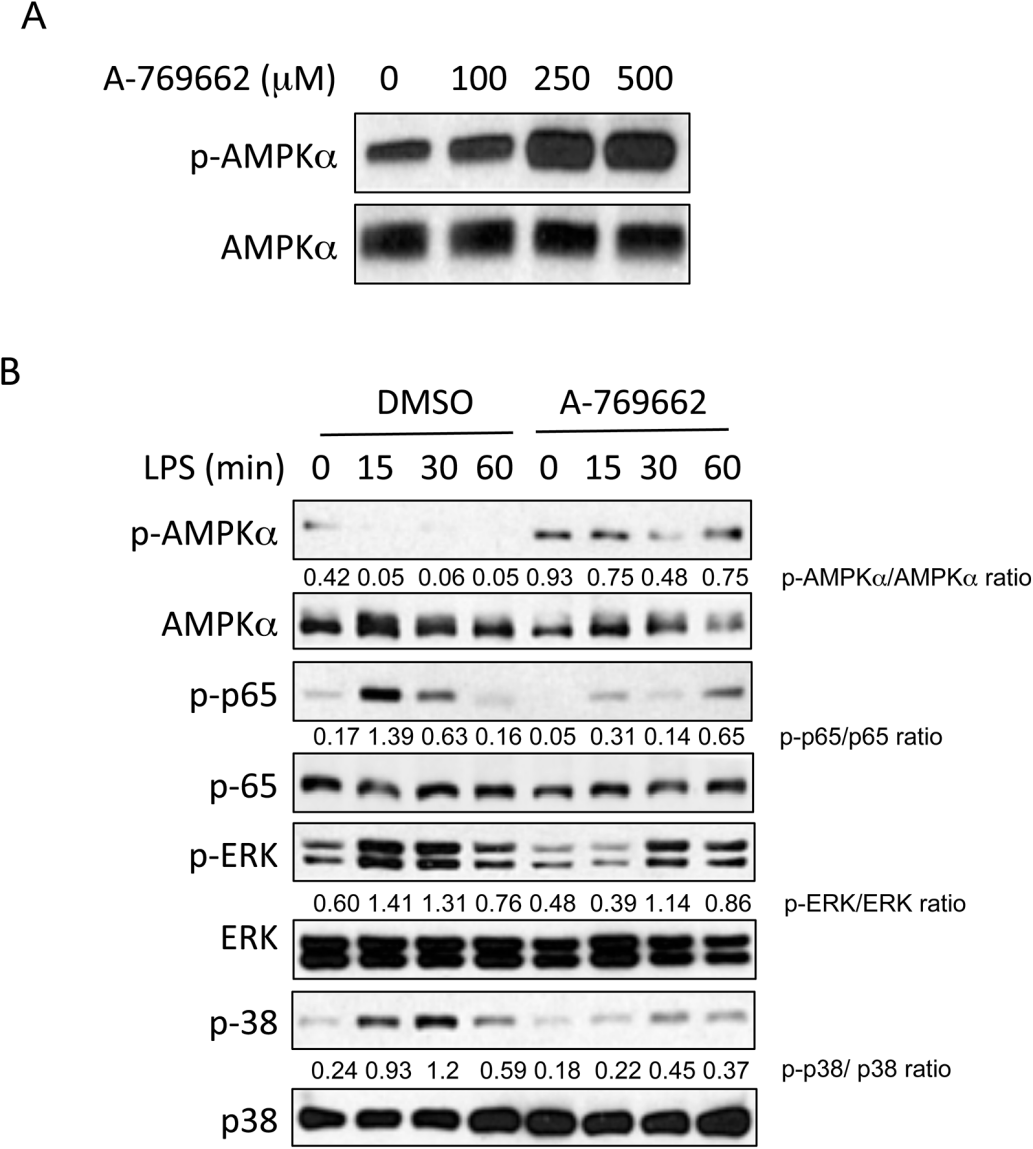
A-769662 activated AMPKα and regulated NF-κB and MAPK signaling in BMDMs. BMDMs were cultured in the presence of different concentrations of A-769662 for 2 hours (A), or BMDMs were pre-treated with A-769662 for 2 hours before stimulated with LPS (1 μg/ml) (B). Lysates of BMDMs were prepared and analyzed for the expression of the indicated proteins by Western blot (WB). Semi-quantitative densitometry analysis of Western blots (arbitrary densitometry units) for phosphorylation of indicated proteins normalized to total protein is shown. Results are representative of three independent experiments.

**Fig 2:**
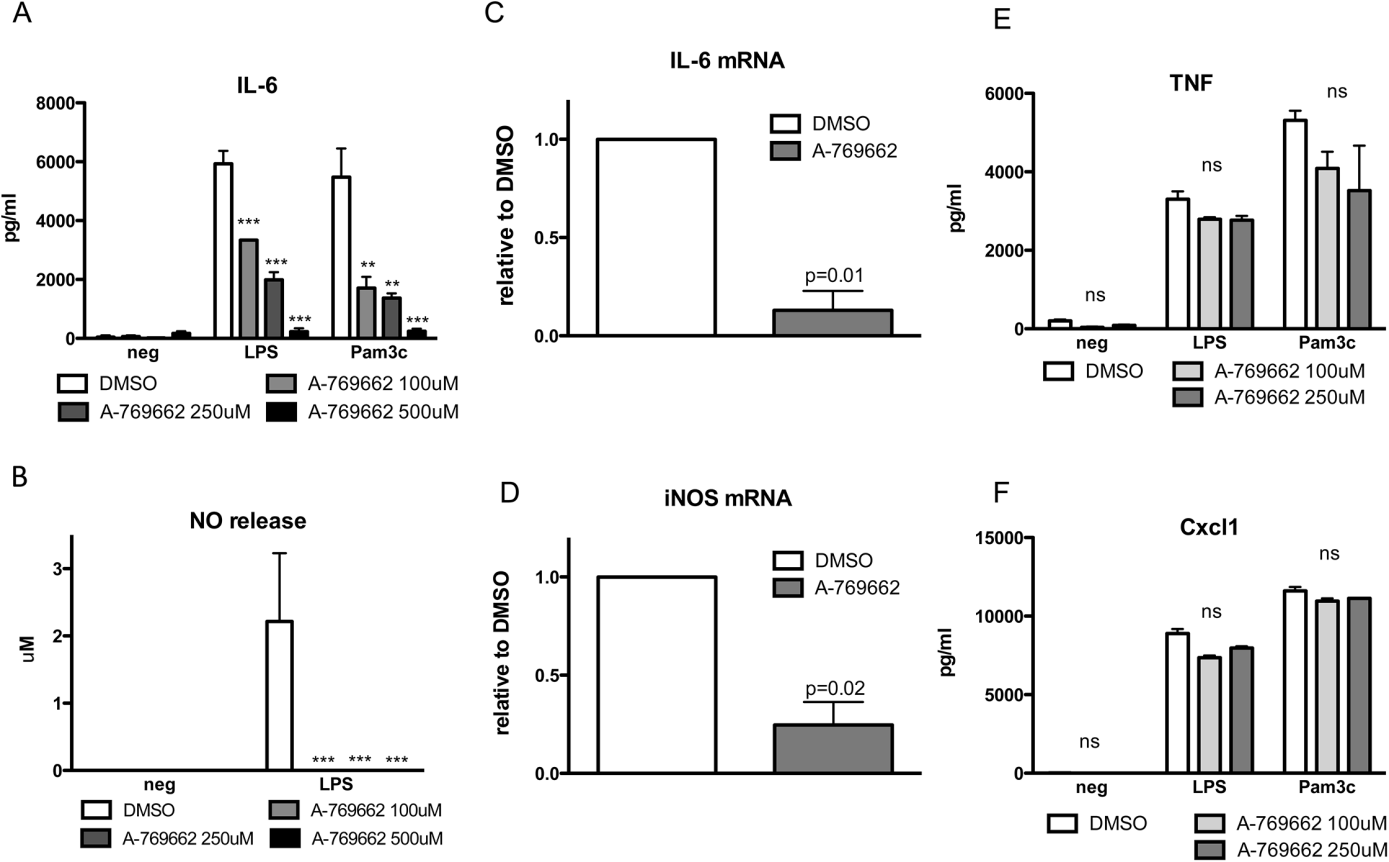
AMPK inhibited IL-6 and NO production in BMDMs stimulated with TLR2 and TLR4 agonists. (A,B) BMDMs were pre-treated with A-769662 (100, 250 or 500 μM) for 2 hours before stimulated with LPS (1 μg/ml) and Pam3Cys4 (1 μg/ml) for 18 hours. The conditioned media was subjected to ELISA and Griess reaction for the indicated cytokine and NO release, respectively. (C,D) BMDMs were pre-treated with A-769662 (100 μM) for 2 hours before stimulated with LPS for two hours. RNA was isolated and analyzed for the expression of the indicated genes. (E,F) BMDMs were pre-treated with A-769662 (100 μM) for 2 hours before stimulated with LPS (1 μg/ml) and Pam3Cys4 (1 μg/ml) for 18 hours. The conditioned media was subjected to ELISA for the indicated cytokines. Results are expressed as means ± SEM.* p< 0.05 vs DMSO; ** p<0.01; *** p<0.001 and are representative of four independent experiments.

As macrophages are considered the principal innate immune effector cells of rheumatoid arthritis, we determined the effect *in vitro* of the specific AMPK pharmacological activator A-769662 on BMDMs. We first evaluated activation of AMPKα in cultured primary BMDMs stimulated with A-769662. As shown in Fig 1A, phosphorylation of AMPKα Thr172 was enhanced by A-769662 at all doses tested. A-769662 (100 μM) was able to inhibit LPS-induced phosphorylation p65 NF-κB, phosphorylation of Erk1/2 MAPK, and phosphorylation of p38 MAPK (Fig 1B and S1 Fig) in BMDM stimulated with LPS. It also inhibited IL-6 secretion of BMDMs stimulated with Pam3Cys4 and LPS (Fig 2A), NO release (Fig 2B) and iNOS and IL-6 mRNA expression (Fig 2C and 2D) after LPS stimulation. However other cytokines such as TNF and chemokines such as CXCL1 were not affected by A-769662 treatment (Fig 2E and 2F). These data suggest that activation of AMPK attenuated inflammatory responses to TLR2 or TLR4 agonists in macrophages *in vitro*.

### A-769662 treatment abrogated joint damage in two murine models of inflammatory arthritis

**Fig 3:**
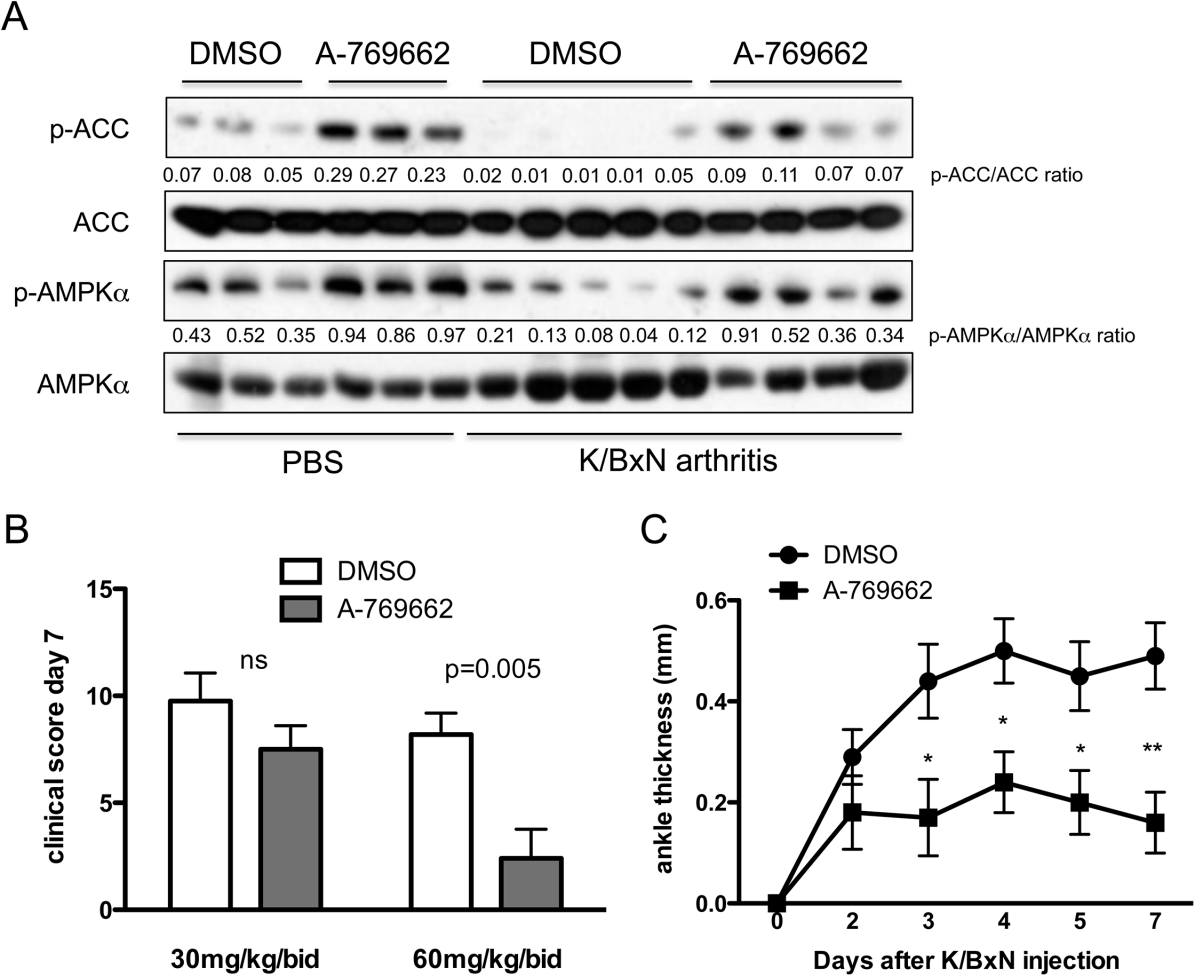
A-769662 treatment activated AMPK in joints and diminished clinical arthritis in passive K/BxN arthritis. WT mice were injected with 150μl serum from adult K/BxN mice on day 0 to induce arthritis. A-769662 was administrated twice a day from day 0. (A) Proteins of joint tissues from naive and arthritic mice injected with control vehicle (DMSO) and A-769662 (60mg/kg) were extracted and analyzed for phospho-AMPKα, phosphor-ACC, total AMPKα and total ACC by WB. D as DMSO and A as A-796662. Semi-quantitative densitometry analysis of Western blots (arbitrary densitometry units) for phosphorylation of AMPKα and ACC normalized to total AMPKα and ACC respectively is shown. (B) Clinical score at day 7 in mice treated with vehicle, A-769662 at high (60mg/kg) and low dose (30mg/kg) (n = 5 mice per group). (C) Ankle thickness in DMSO-treated animals (black circles, n = 9) and high dose A-769662-treated animals (black squares, n = 5) injected with 150 μl of K/BxN serum on day 0. Values are means ± SEM. * = p<0.05; ** = p<0.01.

**Fig 4:**
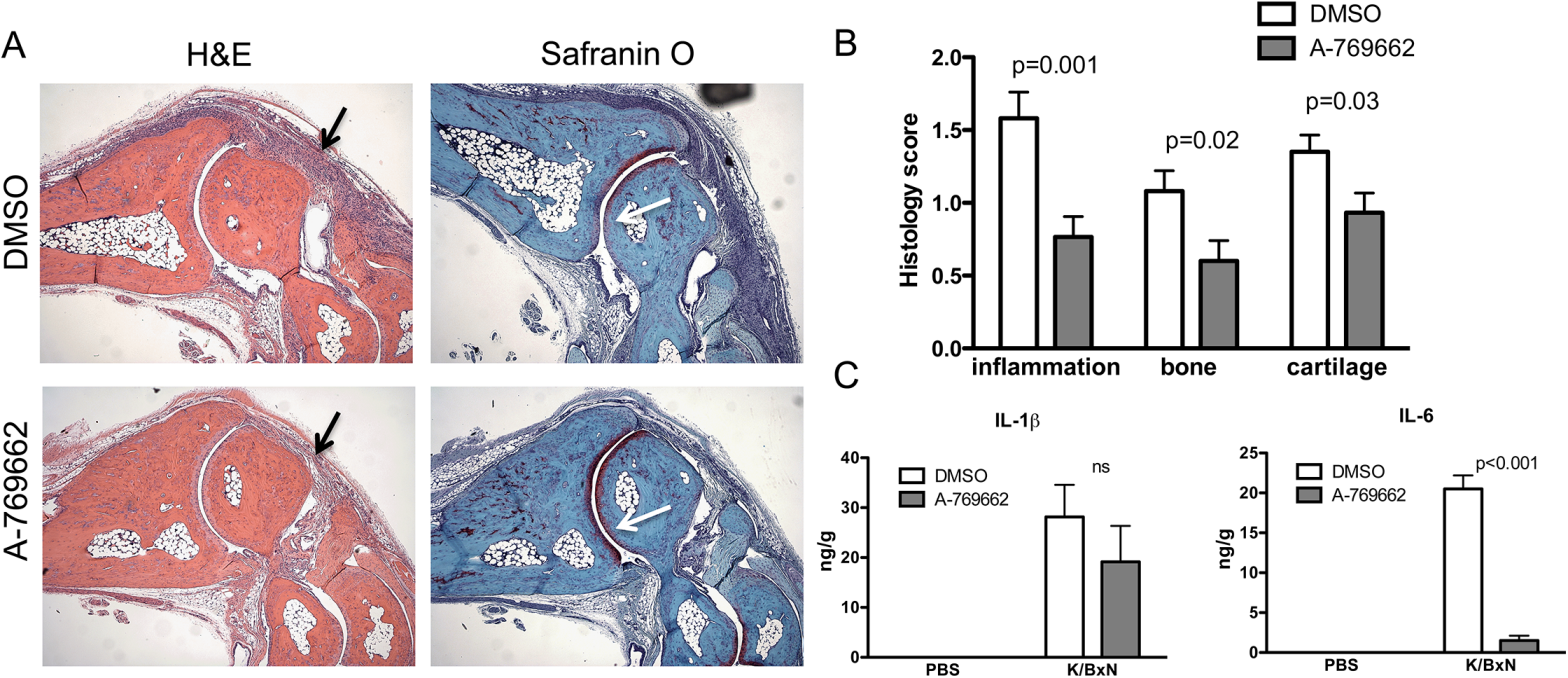
A-769662 treatment abrogated joint damage and IL-6 expression in passive K/BxN arthritis. (A) Representative H&E and Safranin O stained sections of ankle joints on day 7 of arthritis induction in DMSO and A-769662-treated mice. Magnification 200x original. Black arrows in H&E stained sections show joint inflammation and white arrows in Safranin O stained sections show cartilage damage that was reduced in A-769662-treated ankles. (B) Histological scores for joint inflammation, erosion and cartilage damage in vehicle and high dose A-769662-treated mice on day 7 after serum transfer. (C) Proteins of joint tissues from naive (n = 3 mice per group) and arthritic mice (n = 5 mice per group) of DMSO and A-769662-treated mice were extracted and analyzed by ELISA for the presence of the indicated cytokines.

Next, we tested the effect of A-769662 on inflammatory arthritis in mice *in vivo*. We first used the K/BxN passive serum transfer model, which is dependent on innate but not adaptive immunity [13]. We observed that phosphorylation of AMPKα and acetyl-CoA carboxylase (ACC), an AMPK downstream target, in arthritic joint tissues decreased during the peak of inflammation at day 5 (Fig 3A and S2 Fig), but was increased in mice treated with A-769662 (Fig 3A). We used two different doses (30mg/kg/bid and 60mg/kg/bid) of A-769662. As shown in Fig 3B and 3C, higher dose of A-769662 significantly lowered clinical score at day 7, and ankle swelling from day 3. Histopathological analysis at day 7 showed reduced inflammatory cell infiltration, joint destruction and cartilage damage in A-769662-treated mice compared with vehicle-treated controls (Fig 4A and 4B). To evaluate the influence of A-769662 on synovial inflammatory mediators, we determined IL-6 and IL-1β production from these mice on day 5. Clinical score at day 5 was 12 ± 0.4 and 5.5 ± 1.3 for DMSO and A-769662-treated mice (0 = 0.002), respectively. Interestingly, amounts of IL-6, but not IL-1β, were significantly lower in A-769662-treated mice (Fig 4C), suggesting an important role of activation of AMPK in suppression of IL-6 expression.

**Fig 5:**
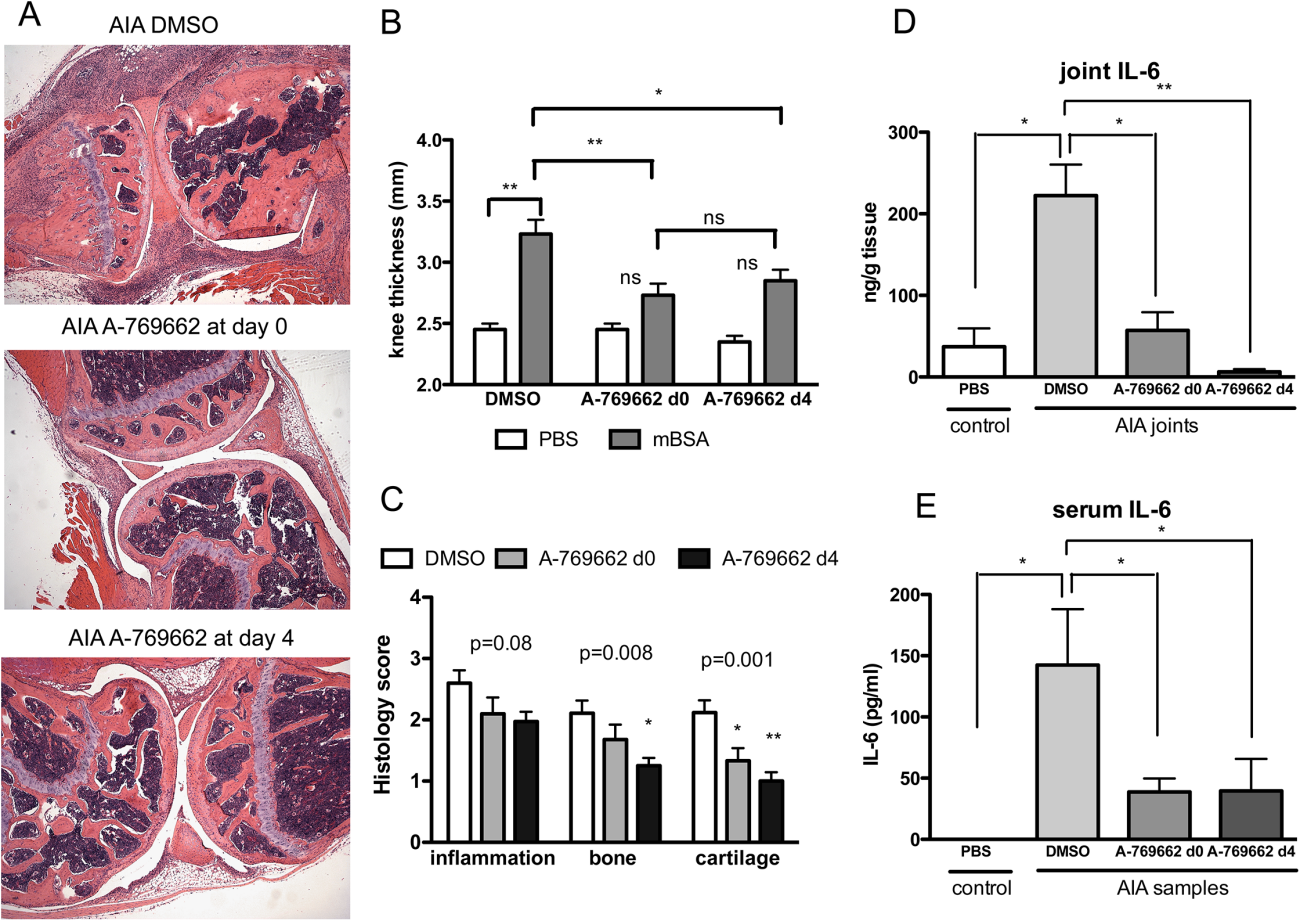
A-769662 treatment also abrogated joint damage and IL-6 expression in AIA. **(**A) Representative H&E and Safranin O stained sections of knee joints on day 10 of AIA induction in vehicle and A-769662-treated mice, which were treated either at day 0 or day 4 after intraarticular injection of mBSA (n = 5 mice per group). Original magnification 200x. (B) Knee thickness in A-769662-treated mice on day 10 of AIA induction. (C) Sections of knee joints were scored for inflammatory infiltration, bone erosion and cartilage damage. A-769662-treated mice had significantly lower scores than WT. (D) Proteins of joint tissues from naive (n = 3 mice per group) and arthritic mice (n = 5 mice per group) of vehicle and A-769662-treated mice were extracted and analyzed by ELISA for the presence of IL-6. (E) Serum from naive (n = 3 mice per group) and arthritic mice (n = 5 mice per group) of vehicle and A-769662-treated mice was analyzed by ELISA for IL-6. Results are expressed as means ± SEM.* p< 0.05 vs WT mice; ** p<0.01

We also studied the effect of the A-769662 in another model of inflammatory arthritis, the antigen induced arthritis (AIA) model, which was shown to be macrophage dependent as well in its effective phase [14]. Clinical and histologic analysis showed a reduction in knee thickness, inflammatory cell infiltration, and significantly decreased joint destruction and cartilage damage in A-769662-treated mice (Fig 5A and 5B). Humoral immunity, tested by the relative levels of mBSA-specific antibodies in sera, was also comparable in all mice treated (data not shown). Importantly, A-769662 treatment not only prevented the onset of arthritis but also successfully suppressed joint damage in mice if treatment was initiated in established disease (Fig 5A and 5B). IL-6 amounts in both joints and serum were also significantly lower in A-769662-treated mice in this model of inflammatory arthritis. (Fig 5C and 5D), confirming the role of AMPK activation in inhibition of IL-6 secretion in inflammation.

### AMPKα1 deficiency mildly enhanced inflammatory arthritis

**Fig 6:**
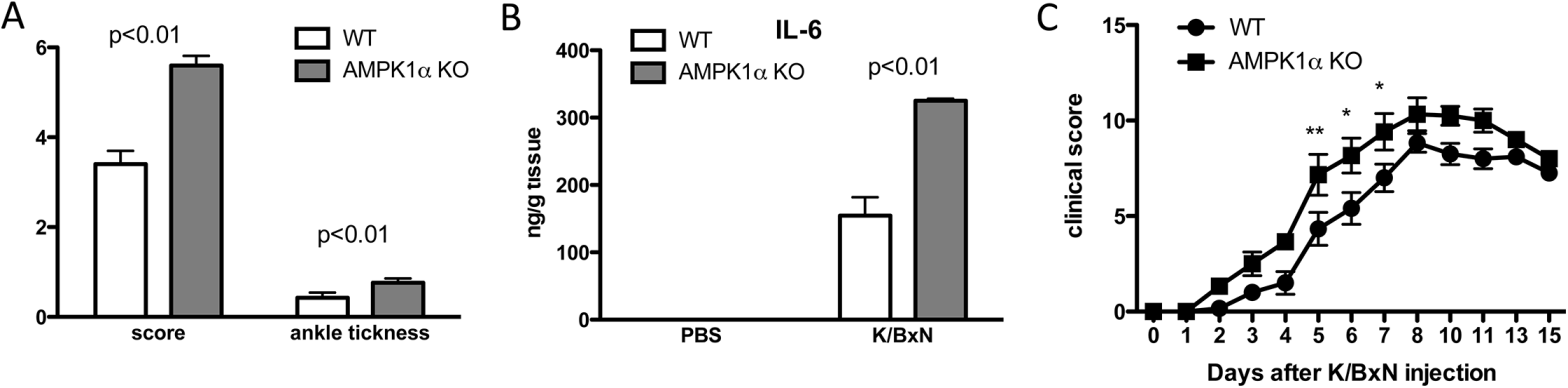
AMPKα1 deficiency mildly increased clinical arthritis in K/BxN arthritis. WT mice and AMPKα1 KO were injected with 100μl serum from adult K/BxN mice on day 0 to induce arthritis. (A) Clinical score and ankle thickness in WT and AMPKα1 deficient animals (n = 5 mice per group) at day 6 after arthritis induction. (B) Proteins of joint tissues from naive (n = 4 mice per group) and arthritic mice (n = 4 mice per group) of WT and AMPKα1 deficient mice were extracted at day 6 after arthritis induction and analyzed by ELISA for the presence of IL-6. (C) Clinical score in WT animals (black circles, n = 6) and AMPKα1 animals (black squares, n = 6). Values are means ± SEM. * = p<0.05; ** = p<0.01.

We finally induced arthritis in AMPKα1 deficient mice. AMPKα1 is the predominant isoform in macrophages. Although clinical scores (score at day 6 was 3.4 ± 0.6 and 5.6 ± 0.48, and ankle thickness was 0.43 ± 0.26 and 0.76 ± 0.21 for WT and AMPKα1 deficient mice respectively, Fig 6A) and amounts of IL-6 in the joints at day 6 (Fig 6B), were increased in the AMPKα1 deficient mice, clinical scores at later time points (Fig 6C) and histologically score at day 8 (data not shown) were not significantly different between WT and AMPKα1 deficient mice, suggesting that inhibition of both AMPKα isoforms may be needed to sufficiently enhance inflammatory arthritis as other cell types, and not only macrophages, may also play an important role in this murine model.

## Discussion

It has long been established that the development of chronic auto-inflammatory conditions, including RA and several cardiovascular diseases, is associated with elevated levels of pro-inflammatory cytokines such as IL-1β, IL-6 and TNF [15–17]. Since AMPK activation was demonstrated to inhibit several pro-inflammatory cytokines in several cell types such as macrophage [18–20], adipocytes [21] and endothelial cells [22], activation of AMPK has been suggested as a target for inflammatory diseases. Furthermore, the AMPK activator AICAR has been demonstrated to attenuate disease progression in several *in vivo* models of inflammation including a rat model of autoimmune encephalomyelitis, a mouse lung injury model and a murine model of colitis [8–10]. Interestingly, metformin, a drug used in the clinic for type 2 diabetes that activates AMPK indirectly, was shown to modestly attenuate inflammation in a murine model of inflammatory arthritis [23]. Given the fact that AMPK activity (indicated by phosphorylation of AMPKα) decreased in macrophages upon pro-inflammatory stimulus (LPS) *in vitro*, and in joints during the peak of inflammatory arthritis *in vivo*, and the ability of A-769662 to prevent inhibition of inflammatory responses, AMPK could be a promising pharmacologic target in arthritis [24].

Most of the agents described as AMPK activators activate AMPK indirectly by inhibiting mitochondrial function (e.g. metformin, berberine), altering cellular AMP levels (e.g. methotrexate), and increasing cellular ZMP, an AMP mimetic nucleotide (e.g. AICAR). AICAR has been found to have AMPK independent effects due to interaction of ZMP with other AMP regulated enzymes and increase of the level of cAMP [25]. Metformin, on the other hand, affects primarily the liver, probably because hepatocytes express the organic cation transporter OCT1, which is needed for metformin transport into cells. Interestingly, immune cells do not express OCT1 transporter [26]. Therefore, there has been a major effort to develop more specific and direct activators, while avoiding some of their side effects, which may be related to inhibition of the respiratory chain rather than to activation of AMPK *per se*. The thienopyridone A-769662 [11] is the first described and certainly the most potent and specific pharmacological activator of AMPK available at present [27]. Although it was shown that AMPKα1 is the dominant AMPKα isoform expressed in macrophages, AMPKα2 has a more prominent role in other cell types that can also been involved in inflammatory arthritis, so activation of both isoforms are preferable. Further research is needed to identify in which type of infiltrating cells in arthritic joints AMPK is activated by A-769662. More important, because of its specificity, A-769662 lacks the off-target effects of AICAR and metformin. In fact, a recent work showed that the majority of these agonists display AMPK-independent effects on several functions with only the synthetic activator A-769662 exerting AMPK-dependent effects on cell proliferation and metabolism [28]. However, we cannot rule out that this drug may also have other activities toward different enzymes in the dosage used in both *in vitro* and *in vivo* experiments as described [29, 30].

A plethora of studies have demonstrated that activation of AMPK suppresses NF-κB signaling, via its downstream mediators including SIRT1, Forkhead box O (FoxO) family, and peroxisome proliferator-activated receptor γ co-activator 1α (PGC-1α) [31], which can subsequently repress the expression of NF-κB-dependent inflammatory factors such as cytokine IL-6. In macrophage cell lines, constitutively active AMPK reduces LPS-stimulated IκBα degradation and subsequent p65 binding to the IL-6 promoter [32]. As murine models of inflammatory arthritis are only partially IL-6 dependent, this could explain why targeting this pathway in murine models with other indirect AMPK agonists in earlier studies had shown only a mild effect. Targeting this pathway in RA seems though an attractive option, as IL-6 is important for both joint destruction and systemic manifestations [33]. Currently, tocilizumab, which binds the IL-6 receptor, is licensed for treatment in active, moderate to severe disease in RA. Elevated lipid profiles with the use of this drug are frequent although more evidence is needed on the long-term cardiovascular safety. Yet, A-769662 was also shown to have beneficial effects on both hepatic steatosis and insulin resistance, thus emphasizing the potential therapeutic implications for AMPK activation in anti-IL-6 responsive RA patients and associated metabolic syndrome [34].

The JNK, p38 and ERK1/2 mitogen-activate protein kinases (MAPKs) pathways also play key roles in inflammatory signaling [35], particularly in rheumatoid arthritis. MAPKs have been implicated as playing important regulatory roles in the production of pro-inflammatory cytokines and downstream signaling events leading to joint inflammation and destruction. A few studies have identified AMPK-dependent suppression of cytokine-stimulated MAPK phosphorylation/activation [3]. For instance, berberine has been demonstrated to inhibit LPS-stimulated JNK, ERK1/2 and p38 MAPK phosphorylation in an AMPK-dependent manner in macrophages [19]. Similarly, A769662 inhibited palmitate-stimulated ERK1/2 phosphorylation in an AMPK-dependent manner in L6 myotubes [36]. Although AICAR has been reported to be associated with enhanced TNF-stimulated JNK and p38 phosphorylation in human colon adenocarcinoma cells [37], in BMDMs both A-769662 and AICAR decrease p38 and ERK-MAPK phosphorylation after TNF induction.

Taken together, our *in vitro* and *in vivo* studies suggest that AMPK activation might be beneficial in inflammatory arthritis by suppressing macrophage inflammatory responses, including IL-6 secretion and activation of NK-κB and MAPK. Therefore, the data provide a rationale for a novel strategy of targeted activation of AMPK as a therapeutic approach in RA.

## Key Messages

- AMPK activation exert significant anti-inflammatory and immunosuppressive effects
- AMPK activation suppressed inflammatory arthritis as well as IL-6 expression in serum and arthritic joints.
- Targeted activation of AMPK might be an effective therapeutic strategy for IL-6 dependent inflammatory arthritis.

## Supporting Information

S1 Fig Semi-quantitative densitometry analysis of Western blots (arbitrary densitometry units) for phosphorylation of indicated proteins normalized to total protein is shown. Results are average of three independent experiments. * p<0.01 vehicle vs A-769662 treated cells.
(TIFF)

S2 Fig Semi-quantitative densitometry analysis of Western blots (arbitrary densitometry units) for phosphorylation of AMPKα and ACC normalized to total AMPKα and ACC respectively is shown. NS: not significant; * p<0.05, ** p<0.01, *** p<0.001.
(TIFF)

## References

[1] O'Neill L.A., Hardie D.G. Metabolism of inflammation limited by AMPK and pseudo-starvation. Nature 2013;493:346–55. 10.1038/nature11862 23325217

[2] Fogarty S., Hardie D.G. Development of protein kinase activators: AMPK as a target in metabolic disorders and cancer. Biochim Biophys Acta 2010;1804:581–91. 10.1016/j.bbapap.2009.09.012 19778642

[3] Salt I.P., Palmer T.M. Exploiting the anti-inflammatory effects of AMP-activated protein kinase activation. Expert Opin Investig Drugs 2012;21:1155–67. 10.1517/13543784.2012.696609 22694351

[4] Muller-Ladner U., Gay R.E., Gay S. Role of nuclear factor kappaB in synovial inflammation. Curr Rheumatol Rep 2002;4:201–7. 12010604 10.1007/s11926-002-0066-1

[5] Hammaker D., Sweeney S., Firestein G.S. Signal transduction networks in rheumatoid arthritis. Ann Rheum Dis 2003;62 Suppl 2:ii86–9. 14532158 10.1136/ard.62.suppl_2.ii86 PMC1766749

[6] Firestein G.S. Evolving concepts of rheumatoid arthritis. Nature 2003;423:356–61. 12748655 10.1038/nature01661

[7] Simmonds R.E., Foxwell B.M. Signalling, inflammation and arthritis: NF-kappaB and its relevance to arthritis and inflammation. Rheumatology (Oxford) 2008;47:584–90. 18234712 10.1093/rheumatology/kem298

[8] Prasad R., Giri S., Nath N., Singh I., Singh A.K. 5-aminoimidazole-4-carboxamide-1-beta-4-ribofuranoside attenuates experimental autoimmune encephalomyelitis via modulation of endothelial-monocyte interaction. J Neurosci Res 2006;84:614–25. 16770773 10.1002/jnr.20953

[9] Zhao X., Zmijewski J.W., Lorne E., Liu G., Park Y.J., Tsuruta Y. et al Activation of AMPK attenuates neutrophil proinflammatory activity and decreases the severity of acute lung injury. Am J Physiol Lung Cell Mol Physiol 2008;295:L497–504. 10.1152/ajplung.90210.2008 18586954 PMC2536800

[10] Bai A., Yong M., Ma A.G., Ma Y., Weiss C.R., Guan Q. et al Novel anti-inflammatory action of 5-aminoimidazole-4-carboxamide ribonucleoside with protective effect in dextran sulfate sodium-induced acute and chronic colitis. J Pharmacol Exp Ther 2010;333:717–25. 10.1124/jpet.109.164954 20237071

[11] Cool B., Zinker B., Chiou W., Kifle L., Cao N., Perham M. et al Identification and characterization of a small molecule AMPK activator that treats key components of type 2 diabetes and the metabolic syndrome. Cell Metab 2006;3:403–16. 16753576 10.1016/j.cmet.2006.05.005

[12] Guma M., Ronacher L., Liu-Bryan R., Takai S., Karin M., Corr M. Caspase 1-independent activation of interleukin-1beta in neutrophil-predominant inflammation. Arthritis Rheum 2009;60:3642–50. 10.1002/art.24959 19950258 PMC2847793

[13] Christianson C.A., Corr M., Yaksh T.L., Svensson C.I. K/BxN serum transfer arthritis as a model of inflammatory joint pain. Methods Mol Biol 2012;851:249–60. 10.1007/978-1-61779-561-9_19 22351097 PMC5426904

[14] Richards P.J., Williams A.S., Goodfellow R.M., Williams B.D. Liposomal clodronate eliminates synovial macrophages, reduces inflammation and ameliorates joint destruction in antigen-induced arthritis. Rheumatology (Oxford) 1999;38:818–25. 10515641 10.1093/rheumatology/38.9.818

[15] Ding C., Cicuttini F., Li J., Jones G. Targeting IL-6 in the treatment of inflammatory and autoimmune diseases. Expert Opin Investig Drugs 2009;18:1457–66. 10.1517/13543780903203789 19715447

[16] Geyer M., Muller-Ladner U. Actual status of antiinterleukin-1 therapies in rheumatic diseases. Curr Opin Rheumatol 2010;22:246–51. 10.1097/BOR.0b013e3283373fa0 20150813

[17] Sethi G., Sung B., Kunnumakkara A.B., Aggarwal B.B. Targeting TNF for Treatment of Cancer and Autoimmunity. Adv Exp Med Biol 2009;647:37–51. 10.1007/978-0-387-89520-8_3 19760065

[18] Galic S., Fullerton M.D., Schertzer J.D., Sikkema S., Marcinko K., Walkley C.R. et al Hematopoietic AMPK beta1 reduces mouse adipose tissue macrophage inflammation and insulin resistance in obesity. J Clin Invest 2011;121:4903–15. 10.1172/JCI58577 22080866 PMC3226000

[19] Jeong H.W., Hsu K.C., Lee J.W., Ham M., Huh J.Y., Shin H.J. et al Berberine suppresses proinflammatory responses through AMPK activation in macrophages. Am J Physiol Endocrinol Metab 2009;296:E955–64. 10.1152/ajpendo.90599.2008 19208854

[20] Sag D., Carling D., Stout R.D., Suttles J. Adenosine 5'-monophosphate-activated protein kinase promotes macrophage polarization to an anti-inflammatory functional phenotype. J Immunol 2008;181:8633–41. 19050283 10.4049/jimmunol.181.12.8633 PMC2756051

[21] Kim Y.D., Kim Y.H., Cho Y.M., Kim D.K., Ahn S.W., Lee J.M. et al Metformin ameliorates IL-6-induced hepatic insulin resistance via induction of orphan nuclear receptor small heterodimer partner (SHP) in mouse models. Diabetologia 2012;55:1482–94. 10.1007/s00125-012-2494-4 22349108

[22] Huang N.L., Chiang S.H., Hsueh C.H., Liang Y.J., Chen Y.J., Lai L.P. Metformin inhibits TNF-alpha-induced IkappaB kinase phosphorylation, IkappaB-alpha degradation and IL-6 production in endothelial cells through PI3K-dependent AMPK phosphorylation. Int J Cardiol 2009;134:169–75. 10.1016/j.ijcard.2008.04.010 18597869

[23] Kang K.Y., Kim Y.K., Yi H., Kim J., Jung H.R., Kim I.J. et al Metformin downregulates Th17 cells differentiation and attenuates murine autoimmune arthritis. Int Immunopharmacol 2013;16:85–92. 10.1016/j.intimp.2013.03.020 23557965

[24] Viollet B., Horman S., Leclerc J., Lantier L., Foretz M., Billaud M. et al AMPK inhibition in health and disease. Crit Rev Biochem Mol Biol 2010;45:276–95. 10.3109/10409238.2010.488215 20522000 PMC3132561

[25] Jhun B.S., Jin Q., Oh Y.T., Kim S.S., Kong Y., Cho Y.H. et al 5-Aminoimidazole-4-carboxamide riboside suppresses lipopolysaccharide-induced TNF-alpha production through inhibition of phosphatidylinositol 3-kinase/Akt activation in RAW 264.7 murine macrophages. Biochem Biophys Res Commun 2004;318:372–80. 15120611 10.1016/j.bbrc.2004.04.035

[26] Koepsell H., Lips K., Volk C. Polyspecific organic cation transporters: structure, function, physiological roles, and biopharmaceutical implications. Pharm Res 2007;24:1227–51. 17473959 10.1007/s11095-007-9254-z

[27] Goransson O., McBride A., Hawley S.A., Ross F.A., Shpiro N., Foretz M. et al Mechanism of action of A-769662, a valuable tool for activation of AMP-activated protein kinase. J Biol Chem 2007;282:32549–60. 17855357 10.1074/jbc.M706536200 PMC2156105

[28] Vincent E.E., Coelho P.P., Blagih J., Griss T., Viollet B., Jones R.G. Differential effects of AMPK agonists on cell growth and metabolism. Oncogene 2014. 10.1038/onc.2014.301 PMC4980123 25241895

[29] Treebak J.T., Birk J.B., Hansen B.F., Olsen G.S., Wojtaszewski J.F. A-769662 activates AMPK beta1-containing complexes but induces glucose uptake through a PI3-kinase-dependent pathway in mouse skeletal muscle. Am J Physiol Cell Physiol 2009;297:C1041–52. 10.1152/ajpcell.00051.2009 19657063

[30] Benziane B., Bjornholm M., Lantier L., Viollet B., Zierath J.R., Chibalin A.V. AMP-activated protein kinase activator A-769662 is an inhibitor of the Na(+)-K(+)-ATPase. Am J Physiol Cell Physiol 2009;297:C1554–66. 10.1152/ajpcell.00010.2009 19828836

[31] Salminen A., Hyttinen J.M., Kaarniranta K. AMP-activated protein kinase inhibits NF-kappaB signaling and inflammation: impact on healthspan and lifespan. J Mol Med (Berl) 2011;89:667–76. 21431325 10.1007/s00109-011-0748-0 PMC3111671

[32] Yang Z., Kahn B.B., Shi H., Xue B.Z. Macrophage alpha1 AMP-activated protein kinase (alpha1AMPK) antagonizes fatty acid-induced inflammation through SIRT1. J Biol Chem 2010;285:19051–9. 10.1074/jbc.M110.123620 20421294 PMC2885183

[33] Md Yusof M.Y., Emery P. Targeting interleukin-6 in rheumatoid arthritis. Drugs 2013;73:341–56. 10.1007/s40265-013-0018-2 23456676

[34] Ferraz-Amaro I., Gonzalez-Juanatey C., Lopez-Mejias R., Riancho-Zarrabeitia L., Gonzalez-Gay M.A. Metabolic syndrome in rheumatoid arthritis. Mediators Inflamm 2013;2013:710928 10.1155/2013/710928 23431244 PMC3572644

[35] Thalhamer T., McGrath M.A., Harnett M.M. MAPKs and their relevance to arthritis and inflammation. Rheumatology (Oxford) 2008;47:409–14. 18187523 10.1093/rheumatology/kem297

[36] Green C.J., Macrae K., Fogarty S., Hardie D.G., Sakamoto K., Hundal H.S. Counter-modulation of fatty acid-induced pro-inflammatory nuclear factor kappaB signalling in rat skeletal muscle cells by AMP-activated protein kinase. The Biochemical journal 2011;435:463–74. 10.1042/BJ20101517 21323644

[37] Su R.Y., Chao Y., Chen T.Y., Huang D.Y., Lin W.W. 5-Aminoimidazole-4-carboxamide riboside sensitizes TRAIL- and TNF{alpha}-induced cytotoxicity in colon cancer cells through AMP-activated protein kinase signaling. Molecular cancer therapeutics 2007;6:1562–71. 17513605 10.1158/1535-7163.MCT-06-0800

