# Supplementary figures and images for: AMPK Activation by A-769662 Controls IL-6 Expression in Inflammatory Arthritis
Source: PLoS One. 2015 Oct 16;10(10):e0140452. doi: 10.1371/journal.pone.0140452 (PMC4608670; doi:10.1371/journal.pone.0140452)

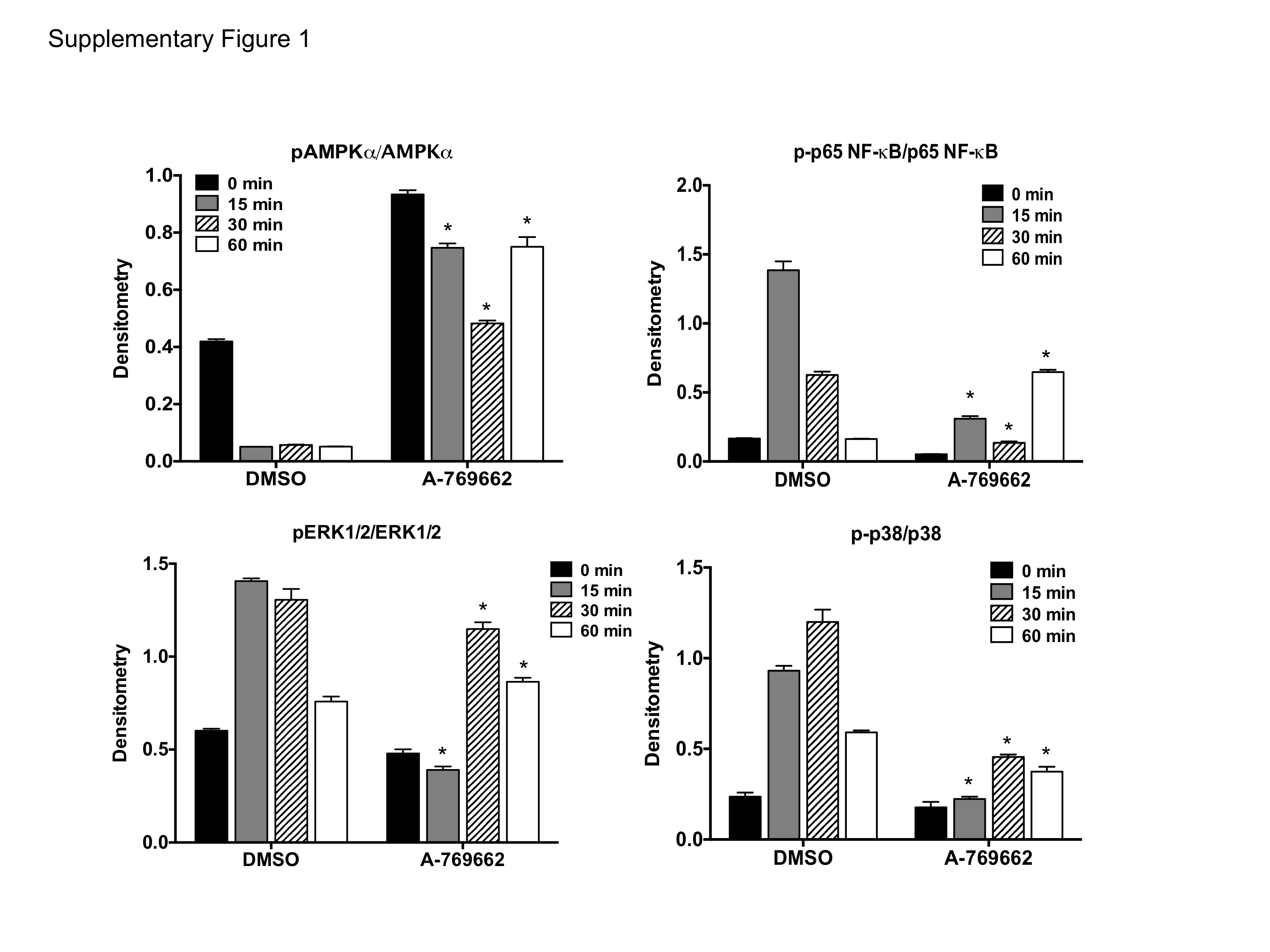

Supplement: S1 Fig — Results are average of three independent experiments. * p<0.01 vehicle vs A-769662 treated cells. (TIFF) [file pone.0140452.s001.tiff]

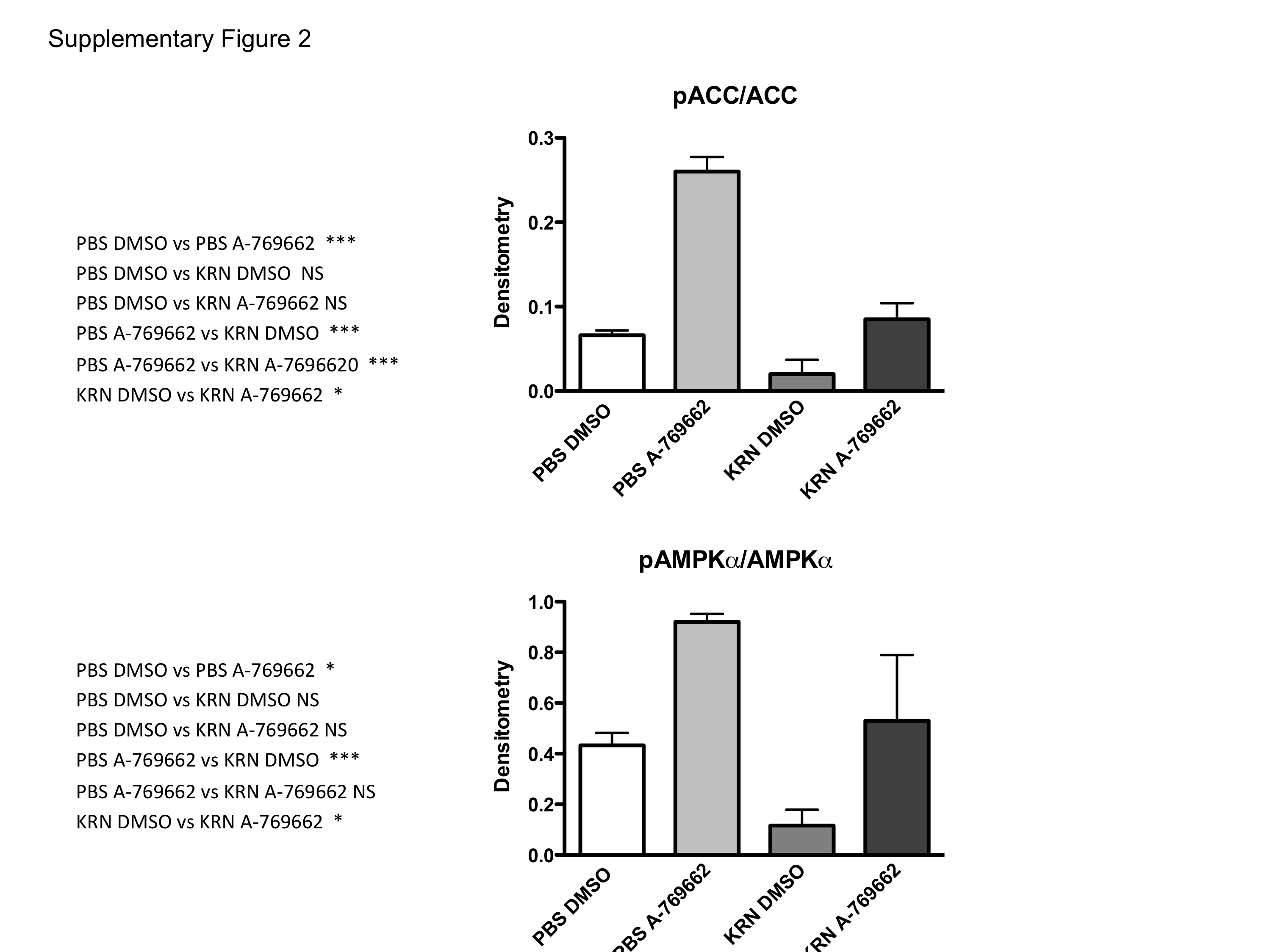

Supplement: S2 Fig — NS: not significant; * p<0.05, ** p<0.01, *** p<0.001. (TIFF) [file pone.0140452.s002.tiff]
